# What Pauline Doesn’t Know: Using Guided Fiction Writing to Educate Health Professionals about Cultural Competence

**DOI:** 10.1007/s10912-016-9430-4

**Published:** 2017-01-07

**Authors:** Lise Saffran

**Affiliations:** Master of Public Health Program, University of Missouri—Columbia, 802 Lewis Hall, Columbia, MO 65203 USA

**Keywords:** Public health, Cultural competency, Health professions education, Health humanities

## Abstract

Research linking reading literary fiction to empathy supports health humanities programs in which reflective writing accompanies close readings of texts, both to explore principles of storytelling (narrative arc and concrete language) and to promote an examination of biases in care. Little attention has been paid to the possible contribution of guided fiction-*writing* in health humanities curricula toward enhancing cultural competence among health professionals, both clinical and community-based. Through an analysis of the short story “Pie Dance” by Molly Giles, juxtaposed with descriptions of specific writing exercises, this paper explains how the demands of writing fiction promote cultural competency.

Molly Giles’s short story, “Pie Dance,” begins with the words, “I don’t know what to do about my husband’s new wife" (1994, 297). At some time in the past, the narrator’s husband left her to marry Pauline, who was his administrative assistant. For some reason, Pauline routinely passes over one toll bridge, one freeway, and two back country roads to linger on the front porch of the narrator’s house and smoke. She declines every invitation to come inside. Even when she’s coughing and is offered first tea, then water, then coffee, then wine, she declines. The narrator speculates that, “If there were to be a thunderstorm—and we often have thunderstorms this time of year—Pauline would have to come in. Or would she?” (302). Pauline’s behavior is a mystery until the very end of the story, when it is revealed that her husband is still sleeping with his ex-wife, the narrator, and is in that very moment hiding in her house. But for most of the story, the mystery is this: why does Pauline go out of her way to sit on the porch of her husband’s ex-wife? Why is she so determined to remain outside?

The close reading of literary fiction has long been a central component in medical humanities programs (Jones 1997), in great part because of its ability to set students puzzling over the complicated motivations of protagonists. The mystery of human behavior is at the root of much in public health and medicine, after all. Why do people do what they do? And because without trouble, there is no story, why, in particular, do people make such unwise, tragic, counter-intuitive choices? Even when they’re smart? Even when they’re mostly good or at least a recognizable mix of good and bad? Why don’t sick people take their medicine, for example? Why do they start smoking? Why don’t they exercise?

Literature is used to awaken the curiosity of those in the health professions, an attribute that the Association of American Medical Colleges identifies as key to the “essential methods and practices of a socially informed and psychologically aware medicine” (2005). Another is empathy (AAMC 2005), and research suggests that engagement with literature promotes medical students’ ability to empathize with patients (McLellan and Jones 1996). Confronting complicated human behavior through literary texts has, along with reflective writing and perspective taking, been associated with patient-centeredness (Blatt et al. 2010), increased self-awareness, and empathy among providers (DasGupta and Charon 2004; Shapiro et al. 2004). Theory of mind research by Kidd and Castano (2013) links empathy to engagement with characters whose “inner lives are rarely easily discerned but warrant exploration” (378).

In “Pie Dance,” the narrator shares with the reader the following when asked about her dog: “I clasp the broom with both hands and gaze fondly at Stray. I am too young to love a dog; at the same time I am beginning to realize that there isn’t that much to love in this world. So when Pauline says, ‘Can it do tricks?’ I try to keep the rush of passion from my eyes; I try to keep my voice down. ‘He can dance,’ I admit” (298). The narrator goes on to describe Pauline as one who, “Favors what the magazines call the ‘layered look’—I suspect because she is ashamed of her bottom. She has thin shoulders and a heavy bottom. Well, I want to tell her, who is not ashamed of their bottom? If not their bottom, their thighs or their breasts, or their wobbly female bellies; who among us is perfect, Pauline?” (300).

The reader recognizes these feelings--loneliness, self-consciousness, compassion--and in so doing gains insight into the characters’ motivations. She suspects that the characters, seemingly so different on the outside, are on the inside not so different than the reader him or herself. After reading a particularly good story, the reader feels she knows the characters in a deep and intimate way. However, some recent literature (Weiner and Auster 2007) has questioned the usefulness, indeed postulated potential harm, in the cultivation of empathy among health professionals. Believing that you understand how another person feels, argue Weiner and Simon, can lead to unfounded assumptions. It can, they claim, short circuit the first attribute on the AAMC list of those to be developed: curiosity (Weiner and Auster 2007). This is a criticism that might have particular resonance in connection with teaching cultural competence where assumptions are often lent additional power by unexplored bias or cultural beliefs.

In this paper I argue that fiction writing builds on the potential of close reading to engender curiosity, while moving students beyond an empathy that is based on presumed knowledge toward a more nuanced empathy grounded in respect. I will demonstrate how Pauline’s story—what she does and does not know—is itself a metaphor for the extra work of imagination and perspective-taking that is required of fiction *writing*, as opposed to the close reading of others’ fiction and how it might offer a particularly useful tool in addressing some of the potential pitfalls of cultural competence education.

In “Pie Dance,” Pauline knows some things; she knows a frozen pie costs one dollar and fifty-six cents. She knows that people can be cruel. But because she refuses to enter the house, she does not know that the broom the narrator keeps on the porch is purely for show. Says the narrator, “I feel no need to clean house, and certainly not with a broom. The rooms at my back are stacked to the rafters with dead flowers and song sheets, stuffed bears and bird nests, junk mail and seashells, but to Pauline, perhaps, my house is vast, scoured, and full of light—to Pauline, perhaps, my house is in order” (300).

The secret that drives much of the two women’s behavior remains hidden from the reader until the end of the story. Throughout, even without knowing why the characters do what they do, the reader affords them the respect of trusting that there is a reason, even if it is hidden. She remains attentive to subtext, alert to what the story might be telling her, such as in the following exchange about Konrad, the man who left the narrator for Pauline:

“Mrs. Dixon, I offer, had a wonderful recipe for blackber…”

“Mrs. Dixon?”

For a second, I almost see Pauline’s eyes. They are small and tired and very angry. Then she tips her head to the sun and the glasses cloud over again.

“Konrad’s mother.”

“Yes,” she says. She lights another cigarette, shakes the match out slowly. “I know.”

“A wonderful recipe for blackberry cake. She used to say that Konrad never liked pie.”

“I know.”

“Just cake.”

“I know.”

“What I found out, Pauline, is that he likes both.” (302–3)

The reader may empathize with the characters’ feelings, she may even suspect that she knows some important things about them, but the *most* important thing, the reason for their actions in the story, remains a mystery until Pauline gets into her car and drives away. Says the narrator:

“Once she turns the corner I drop my hand and bite the knuckles, hard. Then I look back at the house. Konrad steps out, a towel gripped to his waist. He is scowling; angry, I know, because he has spent the last half hour hiding in the shower with the cat litter box and the tortoise.” (304).

And it is here that “Pie Dance” becomes a metaphor for the power of fiction writing to cultivate an empathy tempered by respect and encourages students and practitioners to avoid leaping to early explanations of another’s behavior based on presumed knowledge of her motivations. Health professionals who approach the behavior of patients and communities as if there might be essential pieces of the story that are obscure to them but that make sense from “inside the house” are attentive to subtext, to gesture, to the individual lived experience of the people whose behavior they are trying to understand. Taught to use their imaginations along with their knowledge of social determinants in health, health professionals may be less likely to gloss over important contextual factors and less likely to reach, reflexively, for reductive explanations of behavior.

Evidence suggests that some of the strategies designed to teach respect for cultural difference may paradoxically undermine the curiosity required for the “maintenance of a broad, objective and open attitude toward individuals and their cultures” (Wells 2000) and the ability to view “the individual patient as teacher” (Betancourt et al. 2003). Approaches that rely on educating providers categorically about attitudes, beliefs, and behaviors associated with certain cultural groups, sometimes referred to as the “recipe approach“ have proven to have unintended consequences (Betancourt et al. 2005).^1^They run the risk of reinforcing stereotypes and blurring individual experiences in relation to culture (Saha et al. 2008). Weiner and Simon concur that “a particularly unfortunate consequence of the assumption that empathy provides immediate knowledge of the patient’s state is that it provides a convenient justification for not asking questions—‘If I know what my patient is feeling, I don’t need to ask’—especially the kinds of questions that may get an answer that the physician does not want to deal with” (2007, 125).

The focus on individual experience is one that is central to the creative writing curriculum; zeroing in on the concrete, lived experience of a single individual is the way writers address universal themes. In the words of Ralph Ellison, “All novels are about certain minorities: the individual is a minority. The universal in the novel—and isn’t that what we’re all clamoring for these days?—is reached only through the depiction of the specific man in a specific circumstance" (1955, *The Paris Review*). This perspective echoes the tenets of patient-centered care in medicine, which also promotes an “emphasis on seeing the patient as a unique person" (Beach et al. 2006).

And yet, from a public health perspective, I would add that population data, content knowledge, and theoretical understanding all have important roles to play in addressing the systematic roots of inequality around culture—a goal that is unlikely to be reached through a focus on individual relationships alone. With the growing focus on prevention and population health in medicine (Expert Panel on Cultural Competence Education for Students in Medicine and Public Health 2012; Chockshi 2010), students in the health professions must increasingly understand the role of culture in health and be familiar with a variety of beliefs and practices as they relate to health. As underscored by Betancourt, “cultural competence is not designed to draw attention away from, or address the larger factors that contribute to, racial/ethnic disparities in health, such as poverty, lack of education, the environment, and poor access to care, to name a few" (2006, 500).

Thus, the challenge for cultural competence in health professions education is this: How do we train people who can move back and forth between individual experience and population effects, who can understand how culture influences health outcomes in a global environment while maintaining a perspective of respectful agnosticism with regard to the experiences, perspectives, and beliefs of individuals? An understanding of social context and determinants in health means that we know that the reasons are often a mix of individual, societal, cultural, and economic factors, but knowing that doesn’t tell us which among these factors are relevant for a particular community or a particular patient in a specific situation. At my institution, I teach this through fiction writing in the public health capstone. In spite of all they’ve learned about how social and economic factors influence health behaviors, students’ initial ideas about how to change behavior often begin and end with health education. If the goal is diabetes prevention, their plan is to teach residents to eat better food and encourage them to exercise, often without asking themselves where people in the community will purchase this food and with what resources, or how area streets and sidewalks, work schedules, and transportation options might influence how, when, and if they exercise.

In the first phase, I ask participants to construct a puzzle about human behavior that is particularly mysterious for *them*. Among the modifiable risk behaviors in question, they write down a specific example of one that is especially hard for them to imagine doing personally. Since people are different, I get a range of answers: *Eating Cheetos and drinking orange soda for breakfast. Smoking while pregnant. Smoking at all*. Having chosen the behavior, they then take a mental snapshot of a person—his or her character—engaging in that behavior. At this point, the gulf between students and characters is intentionally wide. Curiosity, however, has been sparked: Why would someone *do* that?

The next phase of the guided fiction writing intervention—in which participants create rounded characters from their snapshot of an individual engaged in an unsympathetic health behavior—would appear to lead directly down the path of assumed knowledge identified by those who raise objections with empathy. In this phase, students answer the kinds of questions that a fiction writer might pose to him or herself during the drafting process. They include the following: *When your character walks into a room full of strangers*, *the first thing she thinks is*…. *Your character*’*s favorite gift on her tenth birthday was*…. *The thing that makes your character feel most hopeful is*…. Designed to give participants a sense of a character’s inner life, this phase often accompanies growing empathy for the character. Though none of the questions deal with the health behavior directly, it is not uncommon for a student to exclaim something along the lines of “I feel like every question here answered why she did it.”

Although the capstone project requires students to consider health issues in the context of a low-income community, students are not required to assign any specific characteristics to their imagined character, yet they often do. Having begun this imaginative exercise with a behavior that is intentionally unsympathetic, student participants draft the outlines of a character who is different from themselves in many other ways as well. Their character may differ from themselves in gender, social or economic class, and race or ethnicity. Taking into account the empathy-related objections, the obvious fear is that having identified an “other,” the student/writer now feels empowered, with a few probing questions, to believe that she understands how that other feels, perceives, and even why she does what she does. How does this phase of the exercise not then interrupt curiosity? How does it, in fact, do the opposite?

First, the intervention in question *is* an act of imagination. Tracing the origin of the word empathy to an early 20^th^-century discussion of art appreciation, Weiner and Simon argue that “[it] is one thing to 'feel into' an inanimate work of art—in the original meaning of empathy—where no direct information is available and error (if such a concept could apply) would have no more significance than perhaps a diminished appreciation of the work” (2007, 124). Of course, the art in question here is not inanimate; it is in fact created by the participant herself. That said, regardless of the ultimate quality of the writing, student participants engaged in this creative writing exercise *are* making art.

The pedagogical approach of this intervention borrows intentionally from the creative writing curriculum; it prioritizes literary craft. Craft elements include concreteness, interior logic, clarity and, perhaps most important, specificity. The requirement of specificity is intended, by its very nature, to lead the student away from generalizations toward a focus on this *particular* invented character. When searching their imaginations for answers to questions such as, “What was the favorite gift that your character received for her tenth birthday?” participants often note that they are both “making things up” and drawing upon their own memories and experiences. In the words of one participant: “I’m having a hard time staying true to fiction.” This, I explain, is what fiction writers *do*, and while it may in part explain the empathy they feel with their characters, they are unlikely to assume they know what the individual received as a gift on her tenth birthday the next time they see someone engaged in the behavior they describe.

So, given that participants never really lose sight of the fact that they are dealing with fictional characters, how does the exercise propose to contribute to cultural competence in the real rather than fictional world? Wouldn’t it be better to have students—and the health professionals they become—encounter the baffling behaviors of patients and communities (behaviors they seek to influence) with their minds entirely free of narratives constructed out of incomplete or even incorrect information about others' motivations? If that were possible, the answer would be yes. But it is not. Human beings do not hold off on interpreting others’ baffling behavior in the absence of complete and accurate data (Baruch 2015; Mønsted et al. 2011). They make assumptions, draw conclusions, and try to figure out what is going on, whether or not the data they have is complete. Clinicians participating in the exercise have noted that in their own encounters with patients, for example, they observe a tendency to interpret a patient’s failure to understand medical advice as a lack of intelligence rather than an issue with health literacy or with the provider’s own ability to communicate (Saffran et al. 2015). The guided fiction writing exercise, therefore, does not ask students as clinicians to construct stories in a vacuum. Rather, its primary purpose is to make participants aware of powerful stories they already tell themselves about health behavior, stories of which they may not even be aware.

As mentioned previously, by the time they enroll in a capstone course, public health students have been exposed to the literature demonstrating that social factors such as economic status, education, and social isolation are more powerful in determining many health outcomes than individual factors such as intelligence or knowledge of a specific risk. Yet, messages about health in wider American cultural and media discourse are often framed primarily in terms of internal factors (Niederdeppe et al. 2011). The fact that the interventions students propose first often focus on individual factors in spite of what they know about the power of social ones would suggest that such popular framing provides a narrative that is particularly difficult to overcome.

Further, when students are given a “golden rule” during the next phase of the intervention, requiring them to write from the assumption that their characters are at least as smart as they are, they frequently find this difficult. Adapted from comments made by David Huddle at the Breadloaf Writers’ Conference, the “golden rule” demands that participants consider and construct coherent internal motivations for their characters’ behavior. The difficulty they often face reveals a tendency—common not only among public health students but beginning fiction writers, as well—to want to reduce “bad” choices to single, easy-to-control, or understandable reasons like lack of intelligence.^2^ Assuming that a character, however flawed, has intelligence equal to one’s own requires accepting also that from his point of view and in his context, the behavior makes sense.

In this phase of the intervention, participants write two scenes: the first involves their character going to work in the morning, and the second involves their character attempting to complete a difficult task with another person. As explained to the students, a scene is a moment in real time, and while it may or may not include dialogue, it includes concrete description, sensory detail, and a focused attention on the character’s surroundings, thoughts, and feelings. It is in the writing of the scenes that participants begin to see their characters as truly worthy of respect. All fiction writing requires imagination, but in a piece of literature, the real imaginative heavy-lifting occurs in the construction of a scene. A scene places the writer first, and then the reader, deeply into the heart of the story. Importantly, scenes are also where the reader and the writer see the interactions between characters in their minds’ eye and, in that seeing, become aware of subtext. Body language, tone of voice, and small or large gestures add layers, and clues, to the mystery at hand. In “Pie Dance,” for example, the sense of the characters’ entanglement deepens in a scene where the two women are interrupted by the arrival of the narrator’s children: “Stray begins to bark and wheel around the garden and a second later the children appear, Letty first, her blonde hair tangled and brambly like mine, then Alicia, brown-eyed like Konrad, and then Sophie, who looks like no one unless—yes—with her small proud head, a bit like Pauline” (303). Scenes are where the story reveals itself to the writer, as well as the reader. This is the point in the writing where my students say things like: *I didn*’*t know until I began writing about her in her house that my character was married*.

Fiction writer and emergency room physician Jay Baruch points out that writing a story is “fundamentally different than reading a story” in that it involves “being present at the beginning, holding each piece in your hand, figuring out what belongs, where and why" (Baruch 2013, 464). The demands of writing fiction require students to call upon memory, sensory detail, perspective-taking, and emotion to enhance their imaginative work, and as a result, an individual’s own biases are often on stark display as are attempts to take short-cuts with one’s thinking. When I was studying fiction writing and a student story would include an absurdly improbable situation—say, a person falling off a boat in Alaskan waters and swimming to shore—one of my teachers would admonish, “You’re not imagining yourself deeply enough into the story.” If the scene is being deeply imagined, the writer realizes that the Alaskan waters are too cold to swim in; if her character falls in, she will be dead within minutes.

Which brings me, finally, to the last attribute, the ability to tolerate uncertainty or ambiguity that is frequently called for in health humanities programs (Bleakely 2015) alongside curiosity, empathy and respect. I don’t know Molly Giles, and I haven’t seen this question addressed in print, but it would not surprise me to learn that she did not begin writing “Pie Dance” knowing that Konrad is hiding in the shower. Though many fiction writers begin with outlines, the creative process requires the writer to be open to surprises. Many writers have only a vague idea where a story is going when they start; the process has often been described as driving a car in heavy fog. The driver can see only as far as the reach of her headlights. Ask any writer, painter, or sculptor––at some point during the creative process the imagination sputters or stalls and any forward momentum seems depleted. The artist has no idea what she should do next. The well runs dry and she has to wait, hoping for it to fill again.

Annie Dillard, in *The Writing Life*, compares the creative process to the life of the inchworm, which she says, “wears out its days in constant panic. Every inchworm I have seen was stuck in long grasses. The wretched inchworm hangs from the side of a grass blade and throws its head around from side to side, seeming to wail. What! No further?” (1989, 7–8). She observes that the inchworm seems to believe, each time, that it will never find another blade of grass until: “its davening, apocalyptic prayers sway the grass head and bump it into something. I have seen it many times. The blind and frantic numbskull makes it off one grass blade and onto another one, which it will climb in virtual hysteria for several hours. Every step brings it to the universe’s rim.” (8).

I suspect panic and hysteria are feelings most health professionals left behind gratefully when, as medical students, they first mapped the anatomy of the endocrine system or as public health students, when they first discovered the power of epidemiology to illuminate seemingly random events. And yet, as a growing consensus of those who train health professionals for the future acknowledge, the ability to tolerate uncertainty is a necessary attribute in a world that, while not immediately ending, is certainly changing at an accelerating rate. Our increased diversity is just one aspect of that change. In a country where an estimated quarter of residents speak a language other than English at home (Koh et al. 2014), change is occurring not just in coastal or urban areas but throughout America. For example, Taney County, Missouri with a population of about 50,000, encompasses a thriving tourist town and regularly hosts seasonal workers from England, Jamaica, Russia, Eastern Europe, Mexico, and Central America. Its residents include growing communities of residents from Mexico, China, India, and Vietnam (personal correspondence with R. Niezgoda).

The benefits of training a more imaginative, empathic, curious, and respectful cadre of medical and health professionals are not restricted to addressing engagement around culture and health disparities, however. As Betancourt et al. write, “Quality improvement of our health care system in these critical areas will improve care not only for minority patients but for all Americans“ (2003, 299). After all, it is not unreasonable to suggest that when it comes to the most private, and even some public, areas of our lives we keep some things hidden, perhaps at times even from ourselves. It is not hard to imagine that in more than one way we may all have someone or something hiding in the shower with the cat litter box and the tortoise.

## Endnotes

- ^1^In studying how providers’ knowledge and perceptions of culture influenced health care received by South Asian immigrant women in Western Canada, Johnson et al. found a tendency to attribute barriers to care to what providers had learned about the cultural beliefs and practices of the underserved group, rather than structural factors and/or discriminatory attitudes. For example, rather than examine barriers such as a lack of female providers or clinic hours, providers described the women as being “shy” and unwilling to seek help except in a crisis (2004).
- ^2^As a participant at the Examined Life Conference at the 2014 Roy J. and Lucille A. Carver College of Medicine commented: “I'm smart enough to not do that behavior so how do I set up a character that is as smart as me but still doesn't get why the behavior is so bad?”
